# On the Use of a Spider’s Web as a Styptic

**Published:** 1867-01

**Authors:** 


*On the use of a Spider's Web as a Styptic.*—On one or two former occasions I have written something on the use of the spider's web as a styptic in cases of excessive hæmorrhage after extracting a tooth. I now wish to add the result of my experience in another case. I do it with the hope and belief that it may be an essential service to some of my professional brethren, and perhaps to some of their patients. It may be thus serviceable on two accounts. First, it can always be obtained, and everywhere, sometimes when other more popular remedies cannot so readily be obtained; and second, because in my hands it has proved efficient where everything else has failed.

About a year ago a man, about eighteen years of age, came to my office to have a lower molar tooth extracted. I examined the tooth, took my forceps and extracted. The operation required rather less force than usual. The tooth came out entire, and clean, and with no laceration of surrounding parts, except the necessary severing of the periosteum. But from the first blood flowed more freely than usual. I directed my patient to rinse his mouth with cold water, which he did considerably longer than the usual time of the flow of blood in such cases, but with no diminution of its flow. I then applied tannin on pledgets of moistened cotton, filling the socket with them. After repeating this application two or three times, the bleeding ceased, and he left. In about three hours after he returned, bleeding as profusely as ever. I then filled the socket from whence the tooth came with cotton saturated with perchloride of iron. This I repeated several times, with a delay of a few minutes between the applications, without any apparent effect. I next applied the persulphate of iron, full strenth, in the same manner, and with no better result. Finally, I procured some spider's web, with which I filled the socket, as I had before done with the cotton, when—I need not say that I was gratified to see—the bleeding stopped almost immediately, and there was no recurrence of it. A. R.—*Dental Cosmos.*

